# Thermomechanical Properties of Carbon and Glass Fiber-Reinforced Epoxy Composites as Function of Drilling Parameters

**DOI:** 10.3390/polym18192316

**Published:** 2026-09-22

**Authors:** Satılmış Ürgün, Sinan Fidan, Eser Yarar, Mehmet İskender Özsoy, Mustafa Özgür Bora

**Affiliations:** 1 Department of Aviation Electrics and Electronics, Faculty of Aeronautics and Astronautics, Kocaeli University, 41001 Kocaeli, Türkiye; urgun@kocaeli.edu.tr; 2 Department of Airframe & Powerplant Maintenance, Faculty of Aeronautics and Astronautics, Kocaeli University, 41001 Kocaeli, Türkiye; sfidan@kocaeli.edu.tr (S.F.); ozgur.bora@kocaeli.edu.tr (M.Ö.B.); 3 Department of Mechanical Engineering, Faculty of Engineering, Kocaeli University, 41380 Kocaeli, Türkiye; eser.yarar@kocaeli.edu.tr; 4 Department of Mechanical Engineering, Faculty of Engineering, Sakarya University, 54050 Sakarya, Türkiye

**Keywords:** polymer–matrix composites, hybrid carbon/glass laminate, drilling, HSS flow step drill, thrust force, delamination, MANOVA

## Abstract

Dry drilling experiments were carried out on pure and hybrid carbon/glass fiber-reinforced epoxy laminates, viz., all carbon (C_8_), all glass (G_8_), glass-faced hybrid (G_2_C_4_G_2_), and carbon-faced hybrid (C_2_G_4_C_2_), using the newly designed high-speed steel (HSS) flow step drill. Drilling operations were conducted at three drill diameters (4, 6, 8 mm), three spindle speeds (125, 250, 535 rpm), and a constant feed rate of 0.1 mm/rev. The thermomechanical behavior and hole-quality assessments were done by means of in-process thrust force monitoring, infrared thermography, 3D optical surface roughness, and scanning electron microscopy (SEM). Drill diameter had a non-linear impact on the thrust force and peak temperature, the highest values of which were obtained with the 6 mm diameter drill, due to its characteristic cutting engagement stages. Both mechanical and thermal behaviors depended on the composite material composition and stacking sequence: the G_8_ laminate had the lowest thrust force and the most dimensionally stable holes, the C_8_ laminate had a low thrust force and minimal thermal reaction, but created exit burrs, whereas hybrid laminates had the maximum thrust force and peak temperature (reaching 103.5 °C) along with the maximum entry/exit asymmetry caused by dissimilar carbon/glass ply interfaces. Spindle speed had little effect on the thrust force and was the main parameter affecting the peak temperature (having a 51.78% contribution). Analysis of variance (ANOVA) and multivariate analysis of variance (MANOVA) revealed the drill diameter, material structure, and their interaction as the significant parameters (*p* < 0.0001) of the coupled thermomechanical drilling response.

## 1. Introduction

With the increasing need for lightweight, strong and damage-tolerant materials, fiber-reinforced polymer (FRP) composite materials have been extensively used in aerospace and automotive industries [1,2,3]. The performance of composite laminate depends on the kind and direction of reinforcing fibers, whereas the use of hybrid fibers in a matrix has proved to be an effective means to develop properties of the laminate by integrating their complementary properties [4,5]. With the integration of these composite laminates in the structures, drilling of holes becomes an essential process because of its significance in forming the holes for the fastening and assembling of composite parts. Hence, machining-induced damages become a matter of concern as they can seriously influence the performance and reliability of composite structures [6,7,8].

The laminates made by carbon and glass fiber-reinforced polymers are highly researched hybrid structural materials due to the possibility of combining the high stiffness and high tensile strength of carbon fibers and the high-impact toughness, relatively low price and damage tolerance of glass fibers [9,10,11]. However, the large difference in elastic modulus, tensile strength, thermal expansion and failure properties of the components may result in different stress states and damage evolution in the laminate [4,12,13]. Therefore, the additional difficulty is presented in processing such hybrid laminates, and the study of the secondary processing and damage mechanisms of carbon/glass hybrids has become a topical issue of research nowadays.

According to Yazman et al. [14], an increased rotation rate leads to decreased cutting force and mechanical damage through thermal softening, whereas feed rate increases axial load. Although the study provides important information on the symmetrical stacking sequence, it is limited by the focus on conventional drilling conditions and does not investigate thermal–mechanical interaction in the laminate during the drilling process. The use of conventional drill geometry restricts the applicability of results to non-conventional stepped drilling tools. According to Murugan et al. [15], drill diameter and stacking pattern affect delamination; in particular, the carbon layer at outer surfaces increases damage resistance. However, the analysis is focused mainly on delamination properties and does not provide comprehensive information about concurrent changes in the thrust force, temperature and 3D shape of holes drilled. Likewise, according to Saghir et al. [16], the machining parameters have a significant impact on surface roughness and delamination when end milling and the diamond tool proves to have a positive effect. However, due to different mechanisms of material removal, contact pattern and axial loading, the conclusions made for end milling cannot be directly applied to drilling operation. Further, Natarajan et al. [17] and Dubey et al. [18] confirmed the role of spindle speed, feed rate, drill geometry and stacking sequence in the control of drilling-induced damage. However, these investigations were mostly performed under conventional drilling conditions using conventional carbide or HSS drills and were mainly focused on delamination and thrust force responses without revealing the exact interrelation between tool geometry, heat generation and entry/exit damage morphology. Moreover, despite examination of several carbon/glass stacking sequences by Dubey et al. [18], the impact of outer ply material on the coupled thermomechanical response has not been fully revealed. Ergene et al. [19] conducted a comprehensive study on drilling performance in anisotropic glass/carbon hybrid laminates by analyzing such factors as thrust force, material removal rate, specific cutting coefficient, delamination and hole geometry. Nevertheless, their study is based on conventional drilling conditions and does not consider the impact of a staged cutting interaction characteristic for flow step drill geometry. Bukhari et al. [20] used the Taguchi and ANOVA methods to investigate the impact of drilling parameters on hole geometry and surface quality. Although the statistical approach successfully determined influential machining parameters, the analysis was limited by selected geometrical and surface quality responses and did not include real-time thermal data and damage mechanisms.

Overall, the aforementioned studies provide valuable insights into the impact of the material microstructure and conventional machining parameters on damage created during drilling of carbon/glass hybrid composite materials. However, there are still some aspects that require further exploration. Namely, there are no systematic studies on the non-linear impact of the flow step drill diameter on the magnitude of thrust force and temperature, on the interaction between staged cutting geometry and different carbon/glass interfaces, and on the interaction between thermomechanical loading and entry/exit hole damage. Moreover, the combined application of thrust force measurement, infrared thermography, three-dimensional optical profilometry, SEM-based analysis of damage and multivariate statistical evaluation is underexplored in the existing literature. Therefore, the purpose of the current study is to fill in these research gaps by investigating the thermomechanical drilling of pure carbon, pure glass and hybrid carbon/glass epoxy laminates with a novel HSS flow step drill.

## 2. Materials and Methods

### 2.1. Materials

Sika Biresin CR80 epoxy resin was used as the matrix material, and the hardener CH80-2 was introduced in the weight ratio of resin to hardener 100:30 (Tekno Endüstriyel Kimyasallar San. Tic. Ltd. Sti., İstanbul, Türkiye). Woven type carbon and glass fibers with the areal weight of 200 g/m^2^ were used as reinforcing materials (Dost Kimya Company (İstanbul, Türkiye). There were four different composite arrangements fabricated using the vacuum infusion method, namely G_8_, pure glass fiber; C_8_, pure carbon fiber; hybrid G_2_C_4_G_2_, with glass fiber on the outer layers; and hybrid C_2_G_4_C_2_, with carbon fiber on the outer layers. The arrangement of the fibers in the laminates is shown in Figure 1. Then, the laminates underwent pre-curing at ambient temperature for 24 h and final curing at 60 °C for 4 h. The thicknesses of the produced C_8_, G_8_, G_2_C_4_G_2_, and C_2_G_4_C_2_ composite laminates are approximately 1.70, 1.84, 1.76, and 1.78 mm, respectively.

### 2.2. Hole-Quality Measurements by 3D Optical Profilometry

Entry and exit surfaces of the drilled holes were studied by a laser profilometer with a chromatic confocal optical pen (Nanovea PS-50, NANOVEA, Irvine, CA, USA). It has a measuring range of 1.1 mm. In the experiment, for every hole, a surface area 12.0 × 12.0 mm^2^ was scanned with step sizes 10 μm along scan axis and 20 μm between successive scans, giving a number of measurement points 1201 and 601 per profile and scan respectively, or 721,801 points in total per surface. Data acquisition was made at frequency 1000 Hz without averaging signal, that corresponds to approximately 23 min scanning time for one surface.

The surface topographies obtained were analyzed through MountainsMap analysis software (standalone version 6.2.7487). It is worth mentioning that the roughness parameters provided in this study do not coincide with the well-known two-dimensional (2D) profile roughness parameters (Ra, Rz, Rmax) according to ISO 4287 [21], which are calculated on a single line profile. Rather, areal (3D) roughness parameters as specified by ISO 25178-2 [22] were used for assessment of the surface texture, which covers the whole measured surface and, therefore, provides the description of the damage around the entire circumferential surface around the hole, and not just a single profile line through the hole center. There were two areal roughness parameters used for characterization of the surface roughness of the drilled holes; Sa, arithmetic mean surface height (analog of Ra) and Sz, maximum surface height, which was the sum of the maximum peak height and the maximum pit depth in the evaluation area (an analog of Rz). In addition to these roughness parameters, the following geometric metrics of the drilled hole were also determined: hole projected surface area (mm^2^), hole volume (mm^3^), and maximum hole depth (µm). The ratio of the projected hole area at the exit to the area at the entry was used as the single parameter index of the dimensional hole quality, values above 1 indicating the hole enlargement due to push-out delamination, while values below 1 were indicative of hole occlusion due to the exit burrs and uncut fibers.

### 2.3. Experimental Setup

The drilling tests (Figure 2) were done in dry cutting conditions for evaluation of the thermomechanical behavior of composite laminates. The dry drilling process was used to simulate real industrial operations in machining of composites, to observe thermal effects and to avoid any influences of the coolant on the structure of the composite laminate. The drills of high-speed steel flow step Ø4 mm, Ø6 mm, and Ø8 mm diameters have been used for the experiments. The HSS FLOWSTEP^®^ drills (DIN 338; RUKO GmbH Präzisionswerkzeuge, Holzgerlingen, Germany) used in the experiments were uncoated and featured a 130°-point angle, a 25–30° helix angle, an h8 diameter tolerance, and a three-face shank. The drills had a normal flute profile and core geometry and were designed for a maximum drilling depth of 5 × D. The machining process was performed using the Toss United TU5032B vertical drilling machine (Nantong Sieg Machinery Co., Ltd., Haimen, China) to provide stable cutting conditions [23]. The experiments were carried out under the constant feed rate equal to 0.1 mm/rev at three spindle speeds of 125 rpm, 250 rpm, and 535 rpm for analysis of interrelations of geometry, kinematics, and architecture of material. The thrust-force data were continuously recorded using a high-precision Kistler 9272 (Kistler, Winterthur, Switzerland) piezoelectric dynamometer, positioned directly beneath the workpiece and connected to a Kistler 5070A charge amplifier (Kistler Instrumente AG, Winterthur, Switzerland), at a sampling frequency of 50 Hz using DynoWare software (version 2825D-02). Processing of signals and control of the thrust force were carried out by means of the DynoWare software 2825D-02. Thermal measurements and heat distribution recording in real time were done by the FLIR A325sc infrared thermal camera (FLIR Systems, Inc.,Wilsonville, OR, USA). The infrared camera was placed at a fixed distance of 50 cm from the working surface of the drill at a 60° angle to provide clear vision of the cutting area [24]. Thermal profiles and temperature fields of the interface between the drill body and workpiece were registered and analyzed by FLIR ResearchIR software (version 1.2.10173.1002).

## 3. Results

This section presents the results of the experimental study on the influence of the material stacking sequence, HSS flow step drill diameter, and spindle speed on the thrust force and heat generation in composite drilling. The complete experimental test matrix along with the output parameters such as thrust force, drill temperature, material temperature, and maximum temperature are listed in Table S1 (Supplementary Materials). The obtained data form the basis for the analysis of the corresponding thermomechanical phenomena, friction interaction dynamics, and energy dissipation processes in pure C_8_ and G_8_ materials as well as in hybrid laminates G_2_C_4_G_2_ and C_2_G_4_C_2_. The subsequent sections present a detailed analysis of the parameters mentioned above.

It should be mentioned that the full test matrix of full factorial design in Table S1 (containing four material laminations, three different drill diameters, and three different spindle speeds) has been used for measuring both forces and temperatures, as well as for the statistical analyses conducted in Section 3.5. However, the specific damage characterization studies such as history of thrust force (Section 3.1), infrared thermal imaging (Section 3.2), optical 3D profilometry (Section 3.3), and SEM damage characterization (Section 3.4) are presented for an intermediate spindle speed of 250 rpm. Such a selection of the spindle speed value as a representative middle point in the factorial ANOVA model is justified by three factors. First, according to the results of factorial ANOVA, spindle speed plays a negligible role in terms of the influence on thrust force (0.37% of variance contribution) and a minor role in terms of hole-quality influence in comparison with material configuration and drill diameter, which are the main contributors of drilling-induced damage. Second, the selection of a constant spindle speed allows us to analyze the influence of drill diameter and stacking sequence on hole quality and damage morphology, which is the subject of this research. Third, optical profilometry is rather time-consuming (about 23 min for scanning one surface); hence, conducting the study only at the middle speed level makes it possible to check all 24 surfaces at the same kinematic conditions.

### 3.1. Effect of Drilling Parameters on Thrust Force

Figure 3 illustrates the variations in the measured thrust force during drilling of C_8_, G_8_, G_2_C_4_G_2_ and C_2_G_4_C_2_ laminates using HSS flow step drills with diameters of 4, 6, and 8 mm at a constant spindle speed of 250 rpm. As seen from the results, the thrust force depends significantly both on drill diameter and laminate configuration, although the dependence on drill diameter is non-linear. Specifically, the drill with the diameter of 6 mm yielded, as a rule, the highest force response among the drills of three diameters. This is especially noteworthy since the flow step drill, unlike a regular twist drill, does not have continuous cutting geometry; therefore, an increase in drill diameter does not always lead to an increase in the corresponding thrust force value. For the C_8_ laminate (Figure 3a), the drill with the diameter of 6 mm yielded the highest force response with instantaneous thrust force values reaching over 120 N during the main drilling process. The drill with the diameter of 4 mm generated significantly smaller forces, while the drill with the diameter of 8 mm produced an intermediate response and had relatively high forces at a longer part of the drilling process. A similar diameter-related tendency was also observed for the G8 laminate (Figure 3b), although with lower force values. Again, in this case the drill with the diameter of 6 mm generated the highest force response reaching about 60–70 N, while the other two drills generated lower force values. Hybrid laminates yielded relatively larger and more fluctuated thrust forces than the G_8_ laminate. Specifically, for G_2_C_4_G_2_ (Figure 3c), the drill of the diameter of 6 mm yielded the force response with peaks reaching about 100 N and sustaining a relatively large value throughout the main material removal stage. The 4 mm drill generated the high force response, while the drill with the diameter of 8 mm generated a lower initial response but maintained relatively large forces during a longer part of the process. For C_2_G_4_C_2_ (Figure 3d), the force response was somewhat irregular—the drill with the diameter of 4 mm generated relatively high forces at the beginning of the drilling stage, while the drill with the diameter of 6 mm demonstrated a repeated high force response, reaching up to about 80–95 N. The drill with the diameter of 8 mm yielded low forces at the beginning of the process but demonstrated some high force peaks during further cutting. First of all, it should be noted that the non-linear impact of the drill diameter on the thrust force response has been observed here: the highest force response was produced, as a rule, by the drill with the diameter of 6 mm rather than the largest one with the diameter of 8 mm. This fact contradicts the general expectation of a proportional relationship between the drill diameter and axial force response. Previously conducted studies on step drilling of composite materials showed that thrust force is greatly affected by geometric features of the stepped cutting profile, including stage ratio and step angle [25]. According to Tsao [25], these factors may greatly influence thrust force generation; hence, a nominal drill diameter might not be sufficient to explain the force response in the step drilling process. Such a relation is more adequate for conventional drilling processes since the material removal is related to a continuous increase in drill diameter. However, in flow step drilling, the process is carried out by engaging the steps of the cut with each layer; hence, effective cutting geometry and interaction between the steps and different composite layers become critical factors for thrust force generation. This explanation is in line with the findings by Feito et al. [26] which state that the stepped cutting geometry leads to a distinct response of the thrust force in step drilling of CFRP laminates and emphasize the impact of the staged cutting geometry on the drilling load. The material configuration is also an influential factor which affects the force response of the process. Indeed, the G_8_ laminate yielded relatively low thrust force values, while hybrid laminates, especially G_2_C_4_G_2_ and C_2_G_4_C_2_, yielded substantially higher values. The C_8_ laminate demonstrated intermediate properties, though with distinct force peaks produced by the drill of the diameter of 6 mm. Higher force levels for hybrid laminates mean that the presence of both carbon and glass fiber layers affects the resistance to the cutting edges during the process. Moreover, differences in the thrust force of G_2_C_4_G_2_ and C_2_G_4_C_2_ laminates show that the stacking sequence might also influence the instantaneous force response, although these laminates contain the same amount of carbon and glass layers. This behavior was previously reported by Gemi et al. [27]; these researchers observed distinct thrust force levels in hybrid carbon/glass composites due to the different arrangement of carbon and glass layers.

### 3.2. Temperature Distribution Analysis

Thermal infrared images during composite drilling under 250 rpm spindle speed conditions are shown in Figure 4. It should be noted that the pure carbon composite C_8_ shows excellent thermal conductivity properties, which provide efficient heat flow from the cutting zone into the tool body. As a result, at a certain value of spindle speed of 250 rpm, the mentioned phenomenon provides a higher drill temperature compared to the temperature of the workpiece, leading to positive thermal gradient ΔT = TDrill − TMaterial = +11.0 °C as seen from Table S1. The described positive difference testifies to the effect of the high thermal conductivity of carbon fibers, which allows channeling of thermal energy away from the workpiece and into the tool. Conversely, the pure composite G_8_ demonstrates poor thermal conductivity, which means the thermal insulation behavior. Thus, the thermal energy generated in the process will remain in the workpiece matrix and the chips flow, providing a negative thermal gradient of about −1.5 °C when the temperature of materials is higher than the drill one. The mentioned result corresponds to the previous studies proving the poor dissipation of heat from the cutting zone due to the low thermal conductivity of GFRP [28]. Hybrid specimens C_2_G_4_C_2_ and G_2_C_4_G_2_ demonstrate high thermal loads with maximum peak temperatures of 103.47 °C and 101.60 °C accordingly (Table S1). Thermographic images shown in Figure 4 prove high heat accumulation zones around the hole periphery in hybrid specimens.

Regarding the impact of drill diameters on Ø4 mm, Ø6 mm, and Ø8 mm HSS flow step tools with a fixed operating speed of 250 rpm, there are several remarkable heat transfer effects that should be considered. Based on traditional cutting theories, with an increase in the drill diameter, the peripheral cutting speed vc = πDn/1000 will also increase; therefore, heat production and its maximal value should be reached for the largest Ø8 mm tool due to enhanced friction energy. Yet, the obtained experimental data differ from this prediction. The intermediate Ø6 mm HSS flow step drill is characterized by the maximal peak value of the average drill temperature 6.3 °C and maximal peak value of the average overall temperature of 102.95 °C (see Table S1), not for the Ø8 mm tool, as predicted. Such thermal behavior corresponds to the data on mechanical properties as well, where the maximal values of thrust force are also reached at Ø6 mm. With the use of Ø8 mm, the maximal overall temperature still remains high (98.23 °C according to Table S1), but the local temperature of the drill tip becomes equal to 55.7 °C. For Ø4 mm, heat generation is mainly localized in the contact area between the drill tip and the workpiece, while in the case of the G8 configuration, a thermal plume rising along the body of the tool is detected (Figure 4). The non-linearity of such thermal effects across diameters is an important empirical fact, which indicates complicated correlations between step geometry, thrust forces, dynamics of interaction, and the thermal mass of HSS flow step tools, which deserve more detailed research. The sensitivity of drilling to step geometry is confirmed by the findings of Chen et al. [29], who found that changes in the cutting-edge diameter ratio of step drills affect composite drilling performance both mechanically and thermally.

### 3.3. Hole-Quality Assessment by 3D Optical Profilometry

The quality of drilled holes was objectively assessed by a non-contact optical 3D profiling technique. The entry (point of tool entry) and exit (point of tool exit) hole surfaces of each specimen were profiled with a scanned area of 12,000 µm × 12,000 µm around the hole, and the obtained topographies were analyzed according to the standard ISO 25178-2 [22]. The following geometrical and roughness characteristics of the holes were determined from the segmented surfaces: hole surface area projection, hole (cavity) volume, maximum and average depth of the holes, and the areal surface roughness parameters Sa and Sz. The entry- and exit-side 3D topography comparisons of the holes for all four laminate combinations in the case of 4 mm, 6 mm, and 8 mm flow step drills are illustrated in Figure 5, Figure 6 and Figure 7. The numerical data of profilometric hole-quality metrics are given in Table 1.

Figure 5 displays a comparison of the 3D topographies of the holes at the entry and exit sides generated using the 4 mm flow step drill. On the entry side (Figure 5a,c,e,g), all the four laminates display a smooth plateau-type topography with a clear circular hole perimeter and a very thin raised ridge due to continuous support of the uppermost plies from the undamaged laminate; hence the vertical measurement range is limited to 672–892 µm. However, at the exit side (Figure 5b,d,f,h), there is a distinct visibility of the woven fabric structure and a systematic increase in the vertical range (up to 920 µm for C_8_ in Figure 5b, 785 µm for G_8_ in Figure 5d, 742 µm for G_2_C_4_G_2_ in Figure 5f, and 1194 µm for C_2_G_4_C_2_ in Figure 5h). There are also protruding uncut fibers, burrs and localized spalls at the hole perimeter. This entry/exit asymmetry is indicative of delamination induced by the axial force of the thrusting action on the exit side where the remaining uncut plies lose the support, deflect under the axial loads of the chisel and step edges, and break in bending rather than undergoing shearing. From the examination of the exit-side topographies, there is evident material dependence: the glass exit plies of G_8_ (Figure 5d) exhibit extensive fiber fuzzing and bundle relief; the glass-skinned hybrid (Figure 5f) displays localized matrix spalling with an increased roughness (Sa = 42.0 up to 113 µm with 2.7 fold increase), whereas the carbon-skinned hybrid (Figure 5h) experiences the highest level of exit delamination among all materials in the 4 mm series, which includes a marked push-out with edge chipping, a vertical range increase (+34%) and a 26% increase in the exit hole area (from 4.91 to 6.20 mm^2^). Additionally, the exit side of C_8_ (Figure 5b) is partially covered with burrs and fiber bundles crossing the hole opening, resulting in a reduced optically measured hole size (from 8.15 to 5.18 mm^2^).

Figure 6 shows the analogous comparison in the case of the 6 mm flow step drill when the highest values of thrust forces in the selected test matrix were achieved (70 N for G_2_C_4_G_2_-D6 and 64 N for C_2_G_4_C_2_-D6, see Table S1). The higher value of the axial force is evident on both hole ends. Again, the pure glass laminate demonstrates the highest symmetries of response in the area of the holes at both entry (Figure 6c) and exit (Figure 6d) sides are almost equal (11.8–12.3 mm^2^), as well as their areal roughness (Sa = 117–112 µm), which is explained by the large failure strain of glass fibers providing bending of the last plies at the exit side. In the case of the carbon laminate, the entry remains smooth and clean; however, its exit becomes substantially rougher (Sa = 63.2–155 µm, an increase by 2.5 times), as well as the damage relief getting deeper (the maximum hole depth increases from 632 to 850 µm). Both hybrid laminates are most sensitive to the increased thrust; thus, the carbon-skinned C_2_G_4_C_2_ already achieves the highest value of entry relief in the whole test matrix (1091 µm in Figure 6g), i.e., bending of the entry-side plies under the higher axial load, and its exit (Figure 6h) enlarges by 22% (from 15.0 to 18.3 mm^2^); moreover, the outer carbon layers are substantially spalled off. On the other hand, the glass-skinned G_2_C_4_G_2_ shows an increase in exit roughness by three times (Sa = 42.1–125 µm in Figure 6f); however, the exit aperture measured optically is diminished (13.5–10.7 mm^2^) due to the uncut carbon fibers spanning the hole mouth.

Results of the comparative analysis of entry and exit damage of the largest tested drill diameter (8 mm) with the most significant cutting engagement are demonstrated in Figure 7. Carbon laminate (Figure 7a,b) shows a combination of a smooth entry (Sa = 46.8 µm) and a roughened exit (Sa = 169 µm, 3.6 times increased), whereas the volume of a cavity rises from 2.57 to 6.32 mm^3^. A slight reduction in the detected size of the exit opening (from 35.9 to 30.7 mm^2^) can be explained by burr obstruction, in accordance with the 4 mm case. In the case of the glass laminate (Figure 7c,d), the structure still represents the most balanced one (Sa = 88.1 to 90.4 µm; area ratio 0.92), but the exit’s maximum depth has doubled (from 394 to 784 µm) due to fiber fuzzing around the hole entrance. The most severe condition in the whole test matrix can be found for the glass-skinned hybrid (Figure 7e,f): this structure already demonstrates the roughest entry (Sa = 182 µm) among all test samples, and, at the exit side, there is an increase in the hole area by 39% (from 23.0 to 31.9 mm^2^), in the cavity volume by more than two times (from 3.87 to 7.90 mm^3^) and the maximum depth equals 976 µm, which proves a significant delamination and material loss in dissimilar layers of the skin/substrate interface. The carbon-skinned hybrid (Figure 7g,h) displays a relatively moderate reaction for such a drill diameter (area decreases from 23.0 to 22.7 mm^2^; Sa = 65.5 to 84.7 µm), which means that the damage mechanism of brittle spallation of the outer carbon plies is more sensitive to the axial thrust concentration of the smaller drills rather than to the larger cutting engagement of the 8 mm drill.

Figure 8 groups the hole-quality complementary parameters into four comparative panels. The cavity volume at the exit of the hole is higher than that at the entrance in 11 out of 12 drilling conditions (Figure 8a). For the pure laminates, the increase is small, e.g., 3.37 to 4.82 mm^3^ for G_8_-D8, whereas for the hybrids the increase can reach 104%, such as in the case of G_2_C_4_G_2_-D8 (3.87 to 7.90 mm^3^) or a fivefold increase, e.g., C_2_G_4_C_2_-D4 (0.423 to 1.99 mm^3^). The maximum hole depth (Figure 8b) increases from entry to exit in 10 out of 12 conditions, the two exceptions being G_8_-D4 (562 to 521 µm) and C_2_G_4_C_2_-D6, where the entrance depth was exaggerated by the high thrust force. Similarly, the areal roughness Sa (Figure 8c) increases at the exit in 10 out of 12 cases, showing the largest increases for C_8_-D8 (46.8 to 169 µm) and G_2_C_4_G_2_-D4 (42.0 to 113 µm); the two exceptions are G_8_-D6 and C_2_G_4_C_2_-D6, which are still within 15% of their initial values. Lastly, the area ratio between the exit and entrance holes (Figure 8d) serves as a one-parameter hole-quality index. While the pure G8 laminate is still the closest to the ideal value of unity for all three diameters (1.00, 1.04, and 0.92), the ratios higher than unity represent an enlarged hole due to the push-out delamination, as is the case for C_2_G_4_C_2_ at 4 and 6 mm (1.26 and 1.22, respectively) and for G_2_C_4_G_2_ at 8 mm (1.39), while those below unity are related to the hole occlusion by exit burrs and uncut fibers, such as for C_8_-D4 (0.64) and G_2_C_4_G_2_-D6 (0.79).

The aggregate results of the profilometric study reveal a very clear pattern of hole quality. Pure laminate G_8_ proved to have the most consistent holes with near unity ratios of exit area to entry area and a minimal increase in the volume of cavities. This result was explained by the higher strain value of glass fibers that allows compensating for exit-side bending of outer plies. The pure C_8_ laminate had good entry surfaces but it showed exit burring and even aperture closure, especially with the smallest diameter drill. Hybrid laminates had the highest degree of entry/exit asymmetry, and the degree of damage increased with an increase in drill diameter. It corresponds with the higher values of thrust forces found for hybrids: interfaces between different plies are easier to delaminate, and stiffness difference between carbon and glass plies allows interlaminar cracking as the flow step drill comes out of the laminate. Maximum exit-side damage in terms of volume (7.90 mm^3^) was recorded in the hybrid laminate with the largest drill diameter (8 mm; G_2_C_4_G_2_-D8).

### 3.4. Evaluation of Damage Mechanism

Figure 9 presents SEM micrographs showing typical damage characteristics in hole-entry and hole-exit surfaces of the investigated C_8_, G_8_, G_2_C_4_G_2_, and C_2_G_4_C_2_ laminates in view of their damage characteristics related to HSS flow step drilling. The SEM images for the C_8_ laminate show distinctive damage characteristics at hole-entry and exit surfaces. Figure 9a demonstrates relatively well-controlled material removal with mostly sheared and fractured carbon fibers close to the cutting path, while the surrounding epoxy matrix is relatively undamaged. The minimal level of damage at the hole-entry surface is caused by the continuous support of underlying plies suppressing peel-up and ply separation under influence of the drill bit. On the other hand, Figure 9b shows significantly higher damage at the hole-exit side in the form of fractured and pulled-out carbon fibers, matrix fragments, fiber bundles along the hole edge, and burrs. As the drill approaches the exit surface, the residual plies lose support and are subjected to bending under the force of axial thrust leading to fiber–matrix debonding and brittle fractures rather than shear fractures. The presence of fiber pull-out, matrix spalling, and fiber bridges at the hole edge is consistent with the enhanced exit surface roughness and reduced optical hole area for the C_8_-D4 specimen and indicates that the failure is dominated by push-out-type damage and burr formation. The hole-entry surface for the G_8_ laminate in Figure 9c shows relatively homogeneous damage due to mostly localized cutting and fracturing of glass fiber bundles with no visible interlaminar separation. The mechanically supported underlying laminate prevents the fiber bending and peel-up during tool insertion. At the hole exit surface shown in Figure 9d, the failure morphology is completely different in terms of extensive glass-fiber fuzzing, fiber pull-out, fractured fiber bundles, and the uncovered matrix at the hole edge. The relatively high failure strain of glass fibers allows significant deformation before the fracture. Thus, fibers undergo bending, stretching and separation from the matrix before being cut by the drill bit. Such a gradual failure mechanism caused by fiber deformation before the fracture contrasts with sudden brittle spalling of the fiber–matrix interface. The pronounced fiber fuzzing and bundle relief shown in Figure 9d are evidence of ductile-like fiber deformation with further fiber separation, fracture, and pull-out in unsupported plies, while relatively equal hole areas indicate that this failure mechanism does not lead to the dimensional hole enlargement in hybrid laminates.

SEM images of glass-skinned G_2_C_4_G_2_ show that the damage pattern related to transition between carbon- and glass-fiber layers is more complex. Figure 9e shows that there is local fiber fracture and matrix removal along with some debonding between fiber and matrix on the entry surface, although the damage is relatively confined because the top glass plies are backed up by the bottom carbon/glass plies. On the exit surface, Figure 9f demonstrates much more intensive matrix fragmentation, fiber pull-out, uncut fibers and local spalling around the hole periphery. Transition from carbon-dominated inner plies to outer glass plies causes heterogeneous damage due to the different stiffness and failure modes of those plies. Under the axial thrust from the flow step drill, such differences cause stress concentration and crack initiation and propagation on interlaminar interfaces and fiber–matrix interfaces, followed by bending and fractures of the unsupported exit plies. As a result, the main mode of exit-side damage is push-out delamination, fiber–matrix debonding, matrix spalling and fiber pull-out. It is consistent with an increase in exit-side roughness and a decrease in the measured aperture of the holes in G_2_C_4_G_2_-D4. The C_2_G_4_C_2_ laminate shows the greatest degree of entry/exit asymmetry at 4 mm diameter drilling conditions as shown in Figure 9g,h. On the entry side, Figure 9g shows localized damage, fracture of outer carbon fibers, together with matrix removal and fiber–matrix debonding. Since carbon fibers have relatively high stiffness and a failure strain lower than that of glass fibers, outer carbon plies are more sensitive to brittle fracture and spalling due to the concentrated axial force applied by the flow step drill. On the exit side, Figure 9h shows very severe damage including intensive fiber fracture and pull-out, matrix fragmentation, edge chipping, uncut fibers and a damaged lip surrounding the hole. Bending of the unsupported final plies caused by the chisel edge and stepped cutting zones, and mismatch of stiffness between carbon and glass plies causes initiation and propagation of interlaminar cracks. Brittle spalling of the carbon-rich outer plies combined with push-out delamination and interfacial debonding causes great exit-side enlargement and higher vertical relief of the C_2_G_4_C_2_-D4. This damage pattern confirms that carbon-skin composite configuration is very sensitive to concentrated thrust loading on the hole exit, where there is insufficient support and unstable fracture. The SEM results in Figure 9 are consistent with damage modes that were experimentally investigated for drilling fiber-reinforced polymer composites. In particular, Gao et al. [30] showed that drilling damage is significantly dependent on the position of plies along the hole thickness and typical modes are exit push-out delamination, entrance peel-up delamination, burrs and fiber pull-out during CFRP drilling. Such a mechanism explains the great difference between the relatively limited damage at the hole entry sides shown in Figure 9a,c,e,g and much more severe fiber pull-out, burrs and push-out-type damage at the exit sides in Figure 9b,d,f,h. Similarly, Ashrafi et al. [31] proved that fiber pull-out geometry depends on the drilling conditions based on SEM analysis. It confirms the current observation that fracture and pull-out of the fibers at the exit side leads to a much more rough and irregular shape of the SEM images. Moreover, Kim et al. [32] found that drilling force and undeformed chip thickness affect the fiber pull-out formation, and changes in drill geometry can affect the area and depth of fiber pull-out. It confirms that the deeper fiber pull-outs can cause rougher surfaces.

Figure 10 presents the scanning electron microscope images of the entry and exit sides of holes drilled in the C_8_, G_8_, G_2_C_4_G_2_, and C_2_G_4_C_2_ laminates with an 8 mm HSS flow step drill. The damage modes observed are similar in nature to those observed in the case of 4 mm holes in Figure 9 but considerably more severe. In the case of the C_8_ laminate, Figure 10a shows a relatively intact entry side with sheared and somewhat frayed carbon fiber bundles embedded in a mostly coherent matrix, whereas Figure 10b demonstrates a highly damaged exit side with long uncut fiber bundles extending out of the drill hole walls, large amounts of matrix debonding, and delamination. Such observations suggest that an increase in drill size results in aggravation of push-out-type delamination and fiber pull-out when unsupported bottom plies of laminate were reached. Similarly in the case of the G_8_ laminate, Figure 10c shows a relatively compact entry side with local glass-fiber fracturing and matrix ridging, whereas Figure 10d depicts considerable fiber fuzzing, bundle splintering, and brush-like fiber protrusions. Such damage modes are characteristic for a ductile bending-fracture mechanism for the glass fiber-reinforced laminates. For the glass-skinned hybrid G_2_C_4_G_2_, Figure 10e illustrates the entrance surface with fiber–matrix debonding and localized resin smear that are signatures of frictional heating on the outer glass layers. Figure 10f reveals an extremely disrupted morphology at the exit surface with fiber bundle fracture and interlaminar cavity opening indicating delamination propagation between the carbon and glass layers. Therefore, this configuration proves to be the one showing the highest damage at the exit within the 8 mm test group. For the carbon-skinned hybrid C_2_G_4_C_2_, Figure 10g illustrates relatively controlled fiber shearing and matrix removal at the entrance, whereas Figure 10h shows the highest damage among all configurations with an 8 mm hole size—with a big exposed void, uncut fiber bundles and delaminated matrix flaps around the hole edge due to the brittle fracture of the outer stiff carbon layers when the effect of the internal stabilizing layers was removed after penetration. Altogether, the entrance surfaces shown in Figure 10a,c,e,g show similar, more constrained shear-dominated damage, whereas the exit surfaces in Figure 10b,d,f,h are defined by push-out delamination, fiber pull-out and matrix fragmentation. The hybrid laminates show the highest level of asymmetry between the entry and exit sides, a trend increasing with hole diameter and correlating with high levels of thrust forces and the volumetric exit damage for the 8 mm holes. In particular, the three damage modes of increased push-out delamination, fiber bundle protrusion, and matrix damage observed at the hole exit in the four laminates (Figure 10b,d,f,h) are consistent with the well-known mode by which the increasingly thin and flexible ligament underneath the advancing drill becomes insufficiently stiff for bending as the drill breaks through the laminate. This leads to the instability of the shear loading condition to bending fracture [33]. This damage mode explains the consistent observation of greater exit side compared to entry side damage in the current 8 mm holes because of the increased drill diameter that lowers the rigidity of the residual ligament beneath the cutting edges at the time of breakthrough. The significant difference between entry and exit damage, and the presence of the open interlaminar crack at the carbon/glass interface of the hybrid laminate G_2_C_4_G_2_ (Figure 10f) are consistent with the observation that the carbon/glass/epoxy interface shows fracture behavior that differs from each of the materials. The difference between the reinforcements leads to a more irregular crack path and localized energy release in the region of dissimilar plies [34]. This effect explains why the transitional zone between carbon and glass plies in the current hybrids provides a preferred location for the delamination growth compared to the failure of each ply. Lastly, the relatively smooth damage of the glass-faced laminate G_8_ (Figure 10d), as opposed to the sharp brittle fracture and interlaminar cavity of the carbon-faced laminate C_2_G_4_C_2_ (Figure 10h), is consistent with the observation that including the glass woven fabric layer at the exit side of the hole lowers the degree of delamination induced during drilling compared to the all-carbon exit layer. This occurs due to the higher strain at the point of failure and the more ductile fracture of glass fibers due to the axial load generated at the moment of drill breakthrough [35].

For the inclusion of the experimental observations from 3D optical profilometry and high-resolution SEM images into a single micromechanical framework, Figure 11 below depicts the schematic sequence of damage mechanism evolution during the drilling process on the entry and exit surfaces for both the pure and hybrid laminate. Structural models show that the hole-entry damage is confined and shear dominated in all cases due to the continuous mechanical confinement imparted by the intact underlying plies that prevent the macroscopic delamination of laminates due to the axial cutting action of the tools. However, the hole breakthrough leads to the catastrophic bending instability regime. Due to the decreasing thickness of the uncut laminate, its bending resistance is reduced drastically, and hence the failure mode shifts from confined shear to catastrophic push-down delamination, interlaminar cracking and fiber–matrix debonding.

The unique failure mechanisms shown in Figure 11 correspond to the inherent thermal–mechanical behavior and failure modes of the constituent reinforcing materials. The all-glass lamina (G_8_ in Figure 11a) possesses a large failure strain for glass fibers and allows considerable ductility, resulting in fiber bend and stretch, which leads to a well-balanced and symmetrical round-hole shape with negligible volume material loss. On the other hand, the all-carbon lamina (C_8_ in Figure 11b) reveals typical brittle failure, including sharp fiber fracture, severe matrix spallation, and exit burr formation due to the brittle behavior and high longitudinal modulus of carbon fibers.

The process of damage development within hybrid composites is characterized by a significantly higher level of complexity (Figure 11c,d), as a result of a high degree of mismatching of mechanical properties, in particular, elastic modulus, coefficient of thermal expansion, and fracture toughness between carbon and glass plies, which play the role in stress transfer and cracking paths. As concerns the carbon-skinned hybrid (C_2_G_4_C_2_, Figure 11c), there is a high tendency of brittle spalling and interlaminar delamination caused by high stresses from thrust loads of the step-drill geometry. There is also a visible formation of interfacial micro-cracks at the interface between carbon and glass plies. As regards the glass-skinned hybrid (G_2_C_4_G_2_, Figure 11d), there occurs severe local failure of the matrix material and significant fiber pull-out in the vicinity of the exit point. Moreover, there is also uncut carbon fiber bridging across the exit area that causes a visual hole occlusion and a decrease in the optical exit diameter. The obtained results allow making a conclusion about the preference of the interlaminar interfaces of hybrids as a path of delamination development.

### 3.5. Factorial Statistical Analysis

In order to determine the relative effect of material arrangement, drill diameter, and spindle speed on the obtained output responses, ANOVA was done in full factorial design for each of the responses (thrust force, drill temperature, material temperature, and maximum temperature) with the relative effect of the different factors and interactions determined in percentages (Table 2). In light of the fact that thrust force and the thermal responses are naturally related as opposed to being independent variables, MANOVA was conducted for the four output responses as a vector (Table 3).

The ANOVA results shown in Table 2 reveal that the force and thermal response parameters depend on different hierarchies. In case of thrust force, the dominant sources of variation are the diameter of drill (33.75%) and material configuration (30.65%), while the spindle speed contribution is negligible (0.37%). Such a hierarchy agrees well with the mechanics of thrust force formation—it depends on the cross-sectional area of the engagement between the stepped cutting edges and the fiber–matrix structure as well as on the mismatch between the stiffness and failure strains of carbon and glass plies rather than on the rotation speed of the tool. Importantly, thrust force demonstrates the maximum three-way interaction term among all considered responses (16.63%) as well as the significant interaction term Material × Drill Diameter (9.19%). This suggests that the dependence of force on the diameter of the drill depends on the particular material configuration that is pierced. Such statistical data provide support to the discussion of the non-monotonic behavior of thrust force in Section 3.1, where the 6 mm drill produces an abnormally high thrust force for the hybrid configurations.

The thermal effects show a relatively balanced influence of material and process parameters, with significantly lower interaction effects. In the case of drill and material temperatures, the material configuration continues to play the main role (31.53% and 35.42%, respectively) due to the different thermal conductivities and friction of carbon and glass fiber laminates. The maximum temperature, on the contrary, is mainly defined by the spindle speed (51.78%) being the greatest single effect out of four, while material configuration (20.18%) and drill diameter (21.80%) play smaller but comparable roles. Thus, while the laminate configuration defines the local thermal behavior at the point of cutting, the total thermal load created in the course of the multi-stage interaction of flow step geometry with the material is mostly dependent on the number of contacts, which depends on the speed of rotation. Low values of the interaction effects for three thermal responses (lower than 10% in comparison with 16.63% in case of thrust force) mean that the heat creation is effectively an additive process depending on the material, diameter and speed, while the thrust force creation inherently implies interactions, just as it is expected for the multi-stage cutting geometry of the flow step drill.

As can be seen from the main effects plots (Figure 12), the findings in Table 2 support a non-monotonic recurring “peak at 6 mm” pattern for all the four responses studied [Figure 12a–d]: the 6 mm flow step drill produces the highest mean response values, while the 4- and 8-mm drills produce lower response values. The recurring pattern for the mechanically and thermally different responses points to a geometric reason behind this effect related to the stepped cutting mechanism: at 4 mm, the involved volume is small; at 8 mm, the step distances spread the cutting load over a bigger area; and at 6 mm, the ratio and order of cutting areas provide the maximum fiber–matrix shearing and frictional contact. The material main effects show an opposite pattern of the relationship between the mechanical and thermal responses [compare Figure 12a with Figure 12b–d]: the G_8_ laminate produces the minimum thrust force, but not the minimum thermal response, while the C_8_ laminate produces the lowest drilling and material temperatures and the highest temperatures, respectively. This corresponds to the difference in properties of the two fibers: the brittle carbon fibers shear locally and produce low heat; the high failure strain glass fibers deform, stretch, and contact the tool cutting edge before breaking and thus produce more frictional heat under smaller axial force. Both hybrids take the highest values among all materials for all the four responses and thus confirm that different-stiffness interfaces increase both the mechanical and thermal loads. Finally, spindle speed produces a relatively flat effect on the thrust force [Figure 12a] and a progressively steeper effect on all three temperatures [Figure 12b–d] because the higher rotational speed means the higher the number of frictional contacts per second with no change in axial cutting force.

The MANOVA results (Table 3) suggest that there is a statistically significant simultaneous influence (*p* < 0.0001) of the factors material configuration, drill diameter, and spindle speed and their second-order interactions on the correlated response vector. The factor material configuration displays the lowest value of Wilks’ Lambda (0.0925) and, thus, the highest value of F (26.09). This corresponds to the highest multivariate effect among these factors. It is explained by the dominating or co-dominating position of the material configuration factor in three out of four univariate responses (Table 2). Stiffness and strain failure of carbon and glass fiber plies affect the mechanical resistance to cutting of these fibers and their friction at the interface with the tool. The factor spindle speed, although being a negligible univariate factor for thrust force (0.37%), occupies second place in the multivariate analysis (Wilks’ Lambda = 0.2294, F = 22.03) as it is the main thermal factor (51.78% for maximum temperature). Thus, the importance of MANOVA is demonstrated—the effects on strongly correlated responses may be underestimated if they are considered separately. Drill diameter appears to be the third significant factor (Wilks’ Lambda = 0.3393, F = 14.52).

The Material × Drill Diameter interaction term displays the highest multivariate interaction effect (Wilks’ Lambda = 0.1451, F = 8.74). This implies that the impact of the drill diameter varies according to the stack arrangement. This is attributed to the interface damage mechanism shown by SEM observations in Section 3.4. In the case of Material × Spindle Speed interaction (Wilks’ Lambda = 0.2601, F = 5.57), a highly significant multivariate effect indicates that the material’s susceptibility to spindle speed changes can be completely explained by its architectural composition. The physical interpretation of the interaction—although keep in mind the physical background—should be made with regard to the big difference between the thermal conductivity of carbon and glass fibers. Indeed, spindle speed defines the frequency of friction contact events at the tool/workpiece interface, and hence it is the primary source of heat. In carbon laminates (e.g., C8), due to higher thermal conductivity, carbon fibers function as an effective heat sink, dissipating friction thermal energy away from the cutting region and into the tool. Hence, with the increase in spindle speed, the corresponding temperature rise is continuously balanced by this mechanism. Glass fibers in G8 laminate and in glass-skinned hybrids (G2C4G2) have lower thermal conductivity and act as thermal insulators. As spindle speed and thus frictional heat flow increases, thermal energy is unable to dissipate quickly and is accumulated inside the low-conducting glass layers and epoxy matrix. Hence, the increase in the thermal accumulation of glass laminates with increasing spindle speed is much more pronounced than in carbon laminates. Therefore, the thermomechanical intensity of the spindle speed effect is directly related to the thermal conductivity property of materials. Finally, the Drill Diameter × Spindle Speed interaction has the least multivariate effect (Wilks’ Lambda = 0.4969, F = 3.99). It means that the cumulative impact of the mentioned factors on the coupled thermomechanical effect is additive. It is confirmed by the insignificant two-way interactions for this factor combination across all four responses (Table 2).

Lastly, the Drill Diameter × Spindle Speed interaction is the one with the smallest multivariate effect (Wilks’ Lambda = 0.4969, F = 3.99). This means that the effect of the combination of these two parameters on the coupled thermomechanical response has an almost additive nature. This conclusion agrees with the small values of two-way interaction effects presented for this parameter pair in Table 2 for all four responses under consideration.

## 4. Conclusions

With regard to the effect of stacking arrangement, material type, diameter of drill hole, and speed of spindle on the drilling characteristics of hybrid carbon/glass fiber composite materials using the novel HSS flow step drill, the following can be concluded based on experiment findings and statistical analysis:

The peak thrust force and temperature occurred at a 6 mm drill diameter for all the laminates investigated. This phenomenon is explained by the multi-step flow geometry of the used drill tool, in which the progressive engagement of steps leads to material removal rather than to the continuous increase in drill diameter; therefore, the nominal drill diameter does not regulate the axial and thermal load.

Material and stacking were found to be the main contributors to variance in thrust force (30.65%) and thermal response (31.5–35.4%). The G_8_ laminate produced the lowest thrust force and showed the most stable dimensional behavior with symmetric holes. The laminate C8 provided low thrust force and the lowest temperatures of drill and material, although it suffered from exit burring and hole occlusion due to exit burr formation. Hybrid laminates (G_2_C_4_G_2_, C_2_G_4_C_2_) provided the highest thrust forces, peak temperatures (103.5 °C) and asymmetrical entry/exit profile with the carbon/glass interface being the preferred delamination/crack initiation point.

The effect of spindle speed was negligible (0.37%) regarding thrust force but was the most significant (51.78%) parameter influencing the maximum temperature, demonstrating that the spindle speed regulates the total accumulated frictional heat generation and not the resistance to cutting.

The quality evaluation of drilled holes by means of 3D optical profiling confirmed the above-described force and thermal effects; the highest volume of damage of the exit-side holes was detected for hybrid laminates (7.90 mm^3^ for G_2_C_4_G_2_ at 8 mm); at the same time, the G_8_ laminate showed the closest exit-to-entry area ratio at each diameter, which makes it the best configuration in terms of hole quality.

Entry-side damage was always local and shear dominated for all laminates, while the exit-side damage was caused by push-out delamination, fiber pull-out and matrix fracture. The severity of exit-side damage increased with the increase in drill diameter and was the highest at dissimilar-ply interfaces of hybrid laminates.

Factorial ANOVA and MANOVA confirmed that material configuration, drill diameter and their interaction significantly (*p* < 0.0001) influence the coupled thermomechanical response of the composite laminates to drilling, while the spindle speed mainly influences the total heat accumulation. Thus, for the HSS flow step drill the most critical drilling parameters are the 6 mm drill diameter and hybrid material configuration, while the pure glass laminate under a controlled spindle speed provides the most favorable drilling conditions.

## Figures and Tables

**Figure 1:**
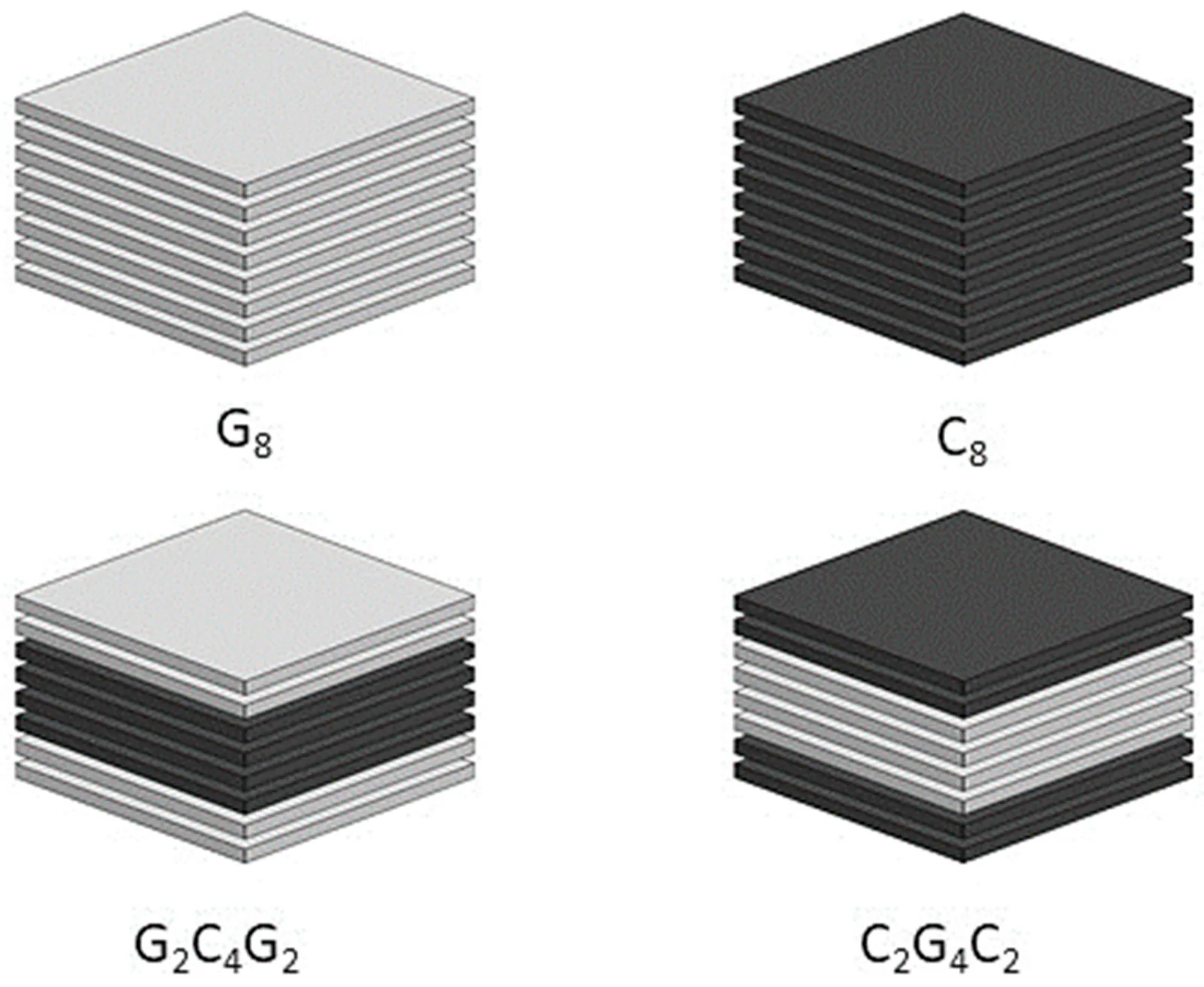
Fiber sequence of the composite laminates.

**Figure 2:**
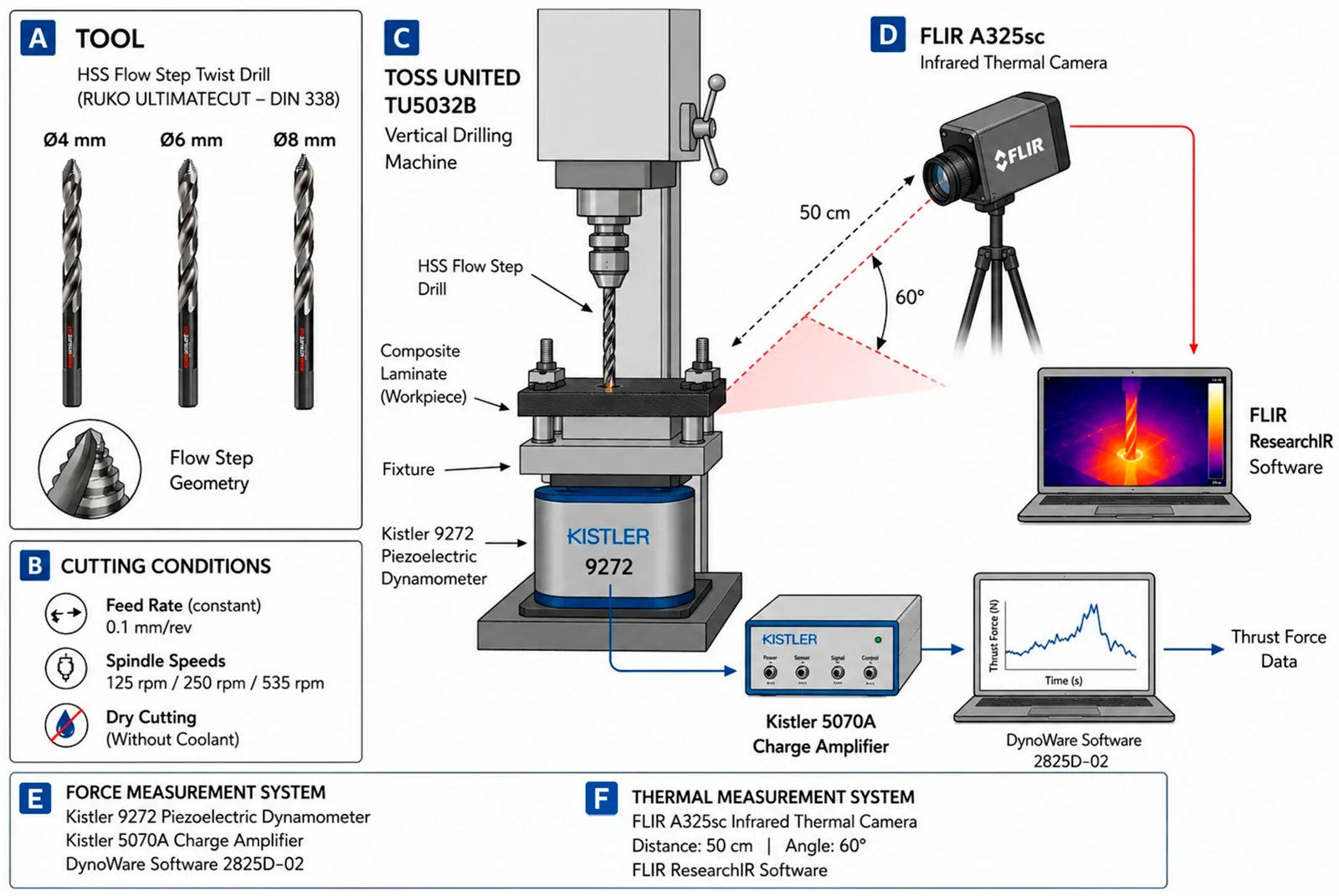
Schematic representation of the experimental setup.

**Figure 3:**
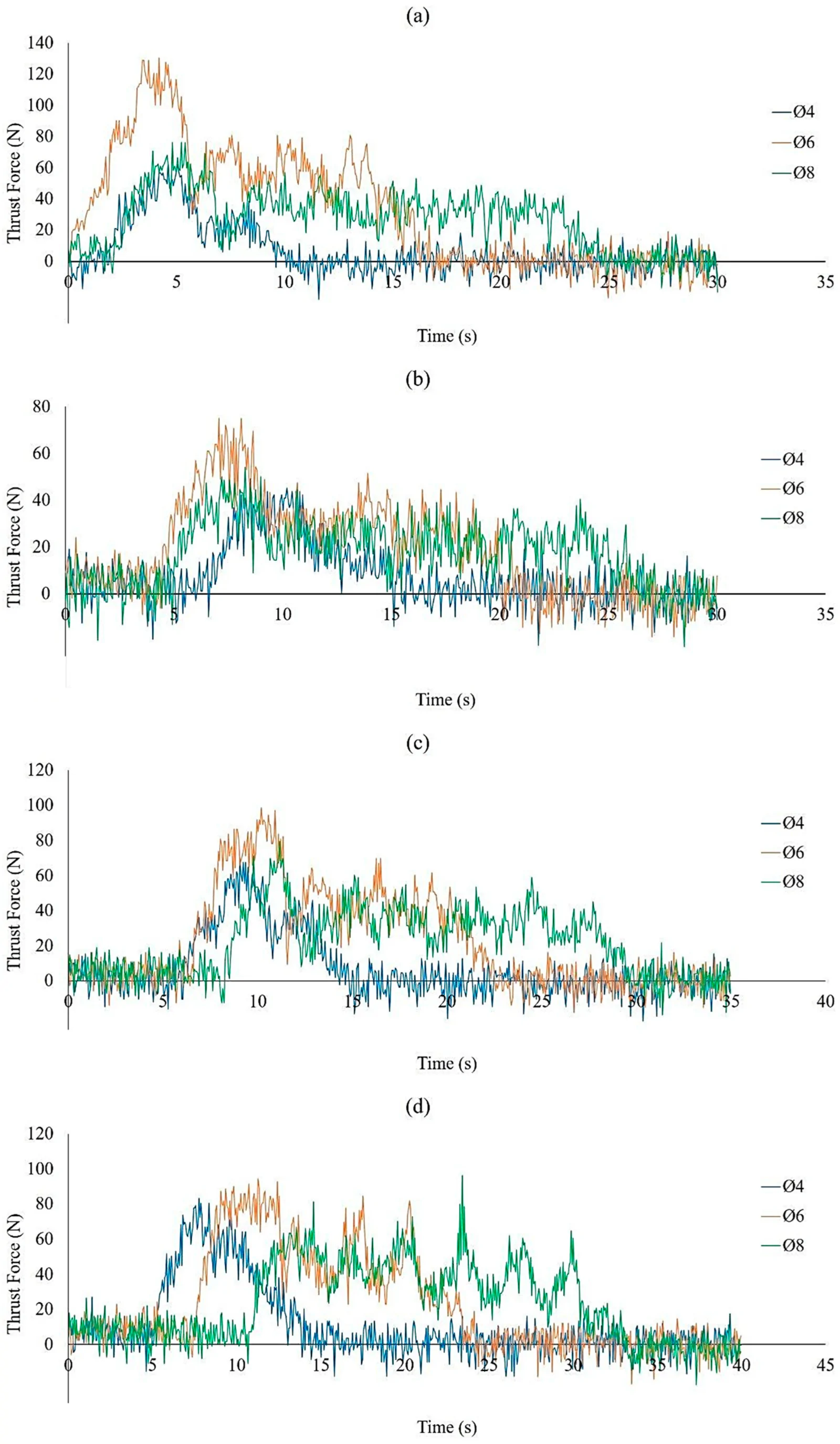
Representative single-trial thrust-force profiles for different laminates drilled at 250 rpm with 4, 6, and 8 mm HSS flow step drills: (**a**) C_8_, (**b**) G_8,_ (**c**) G_2_C_4_G_2_, and (**d**) C_2_G_4_C_2_.

**Figure 4:**
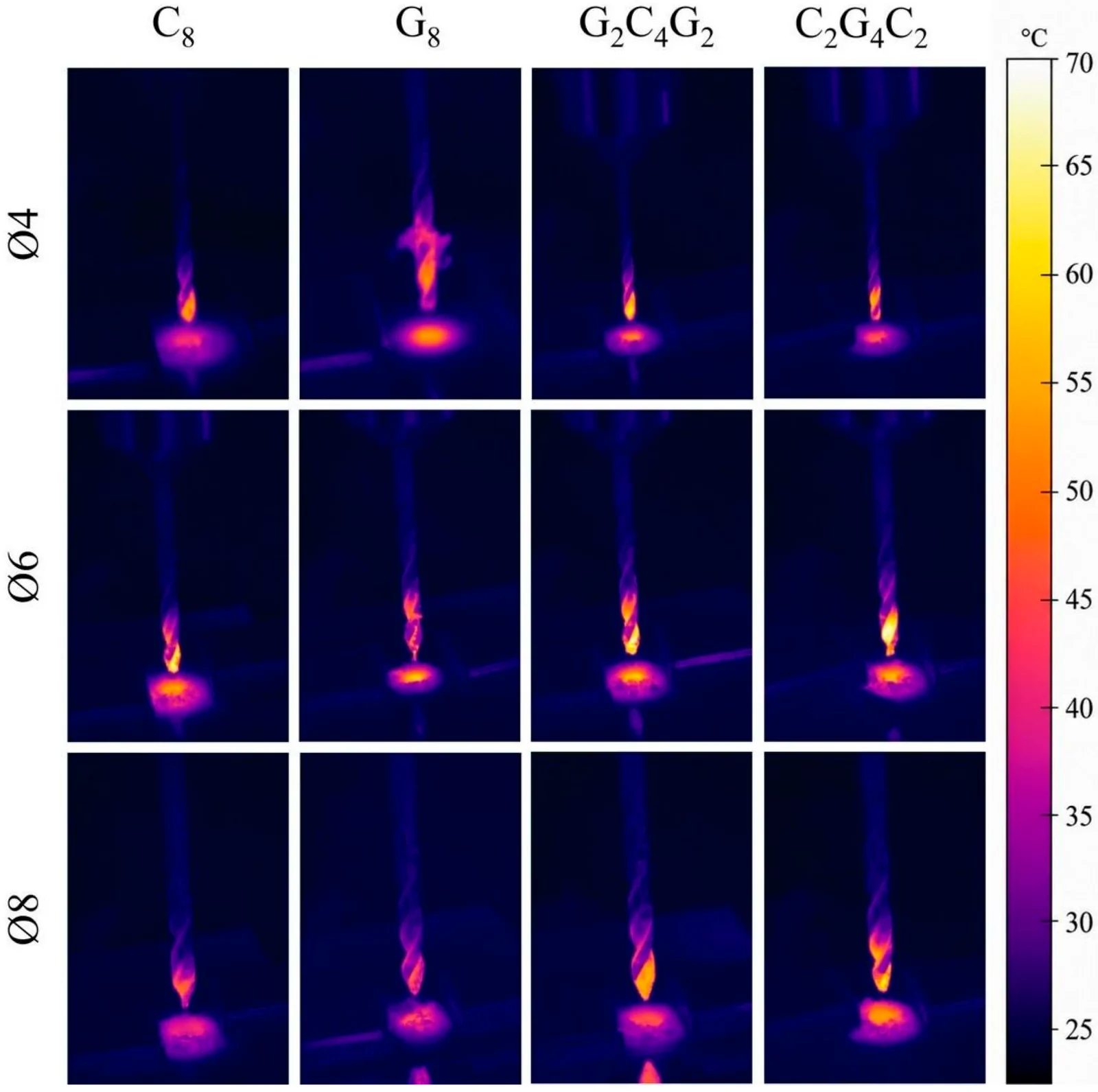
Thermal infrared imagery of composite laminates drilled with HSS flow step tools of various diameters under constant spindle speed (250 rpm).

**Figure 5:**
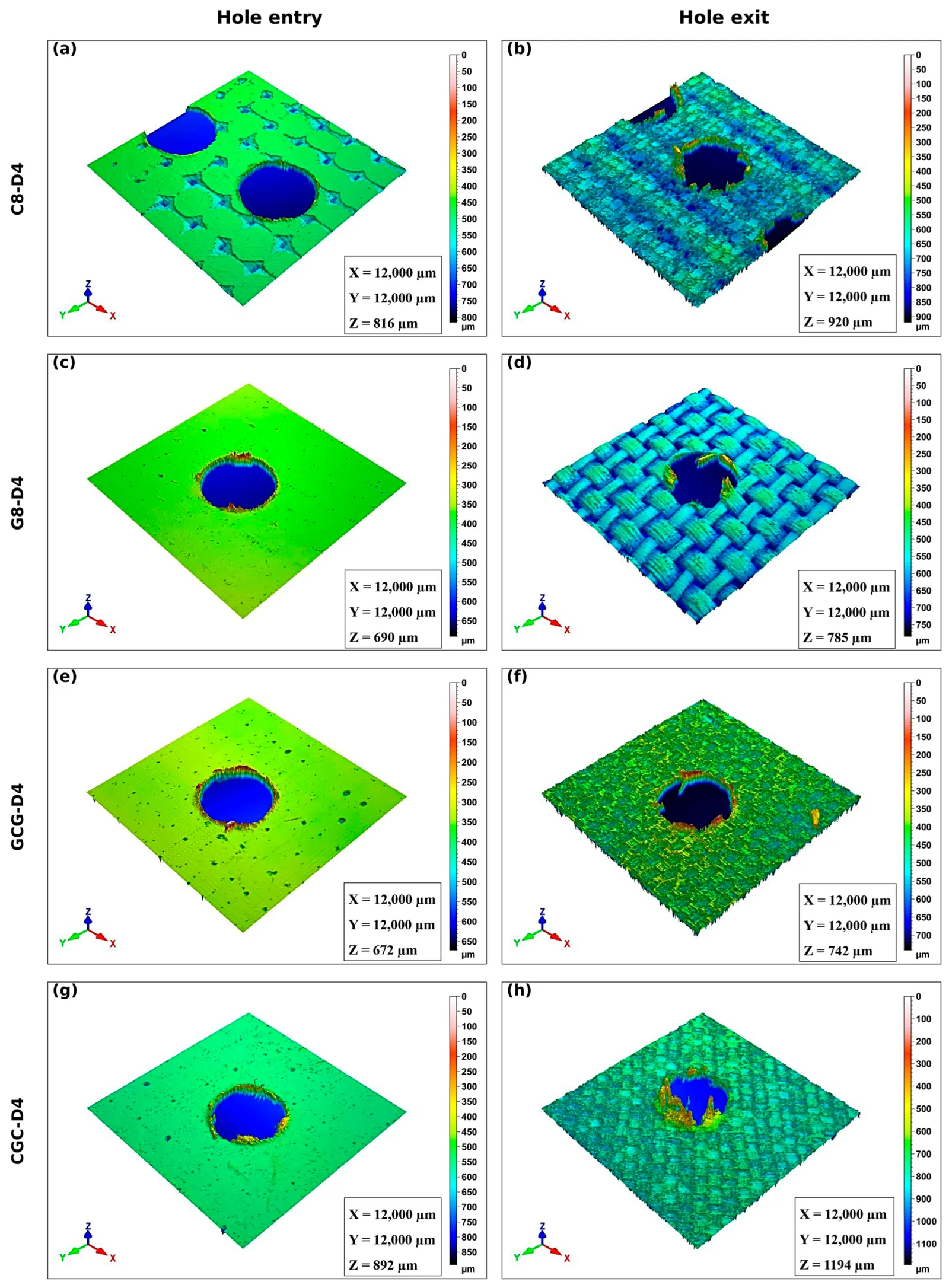
3D surface topographies of the hole entry (**left column**) and hole exit (**right column**) surfaces drilled with the 4 mm flow step drill: (**a**,**b**) C_8_, (**c**,**d**) G_8_, (**e**,**f**) G_2_C_4_G_2_, (**g**,**h**) C_2_G_4_C_2_.

**Figure 6:**
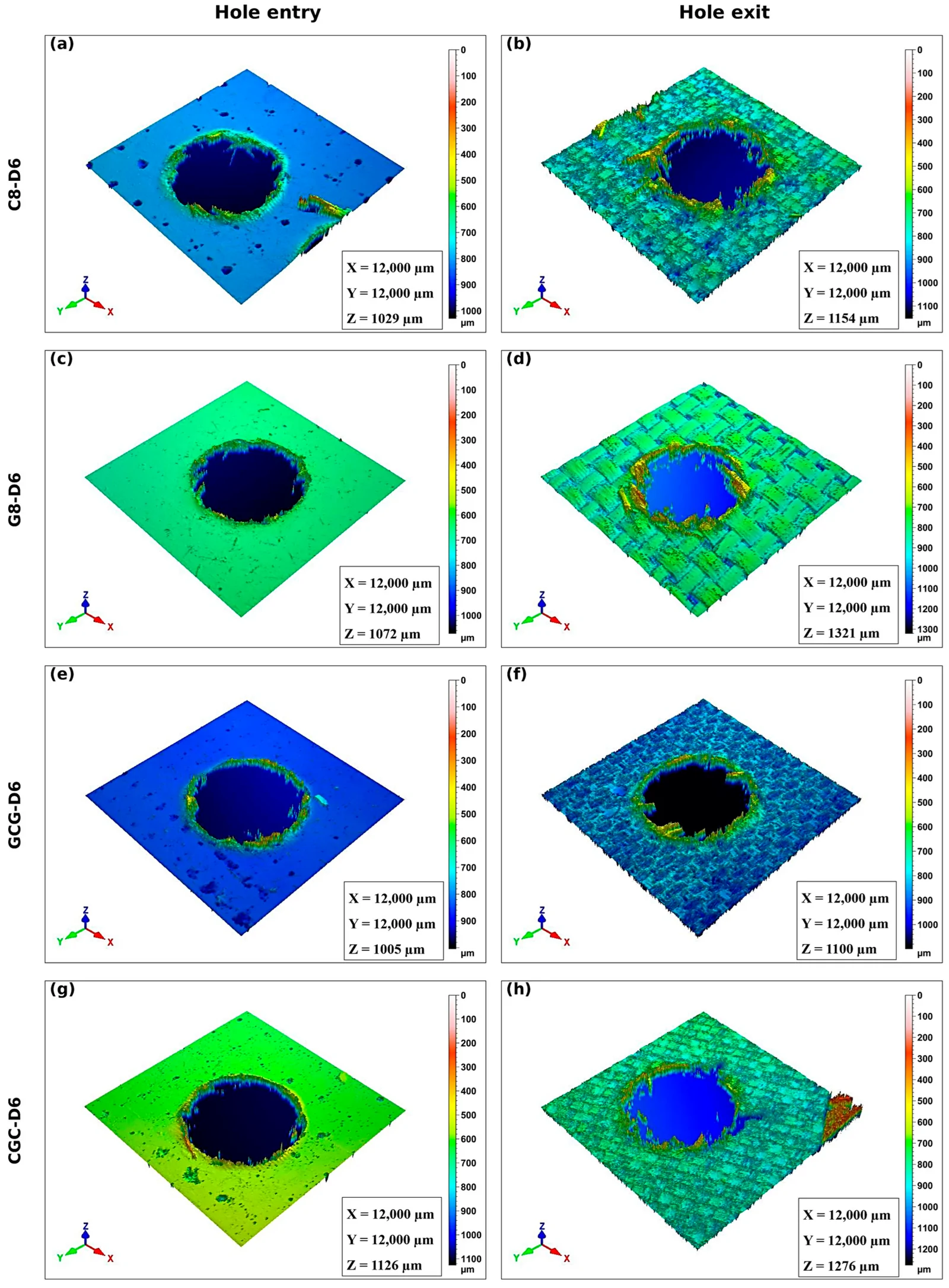
3D surface topographies of the hole entry (**left column**) and hole exit (**right column**) surfaces drilled with the 6 mm flow step drill: (**a**,**b**) C_8_, (**c**,**d**) G_8_, (**e**,**f**) G_2_C_4_G_2_, (**g**,**h**) C_2_G_4_C_2_.

**Figure 7:**
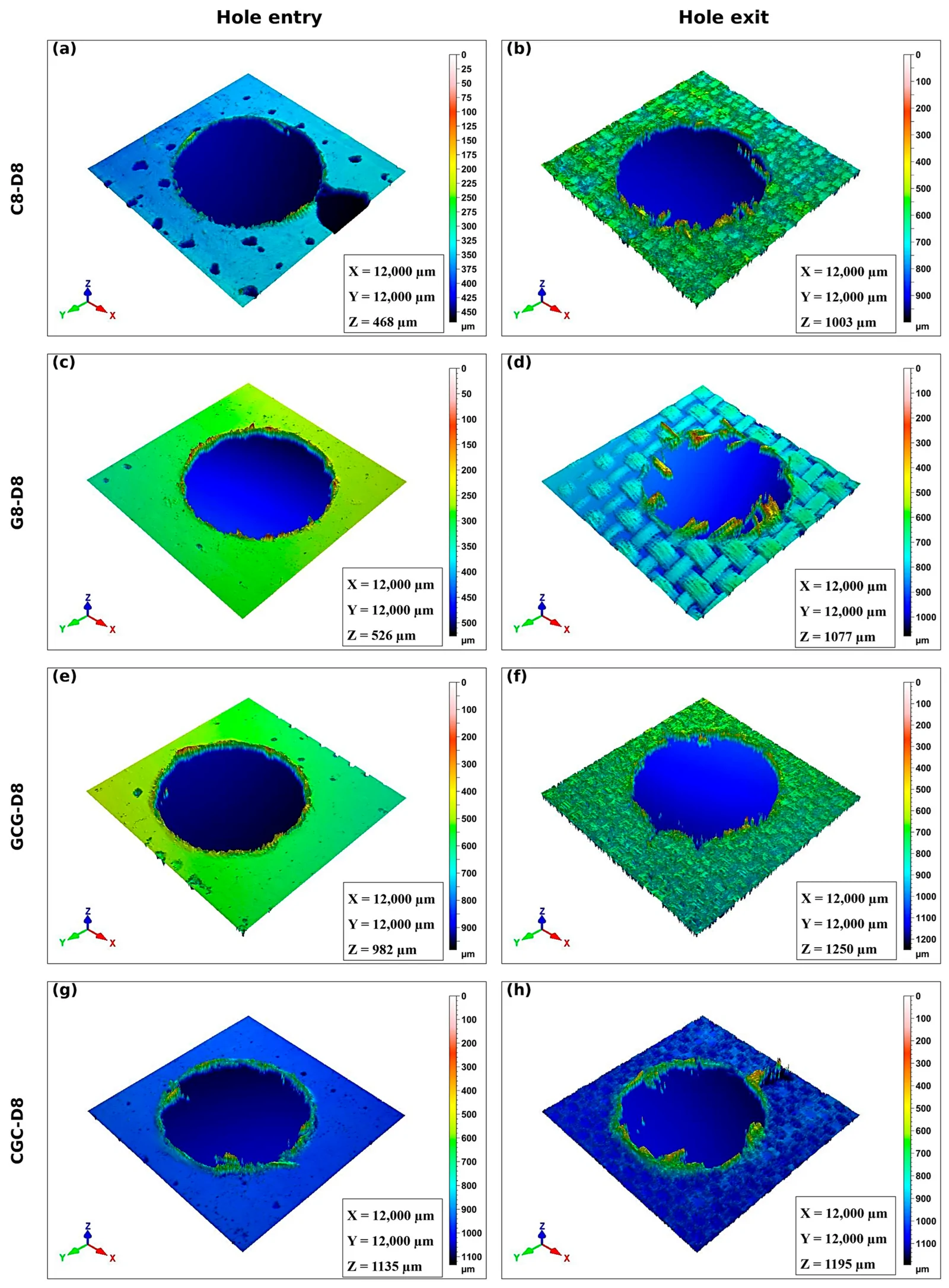
3D surface topographies of the hole entry (**left column**) and hole exit (**right column**) surfaces drilled with the 8 mm flow step drill: (**a**,**b**) C_8_, (**c**,**d**) G_8_, (**e**,**f**) G_2_C_4_G_2_, (**g**,**h**) C_2_G_4_C_2_.

**Figure 8:**
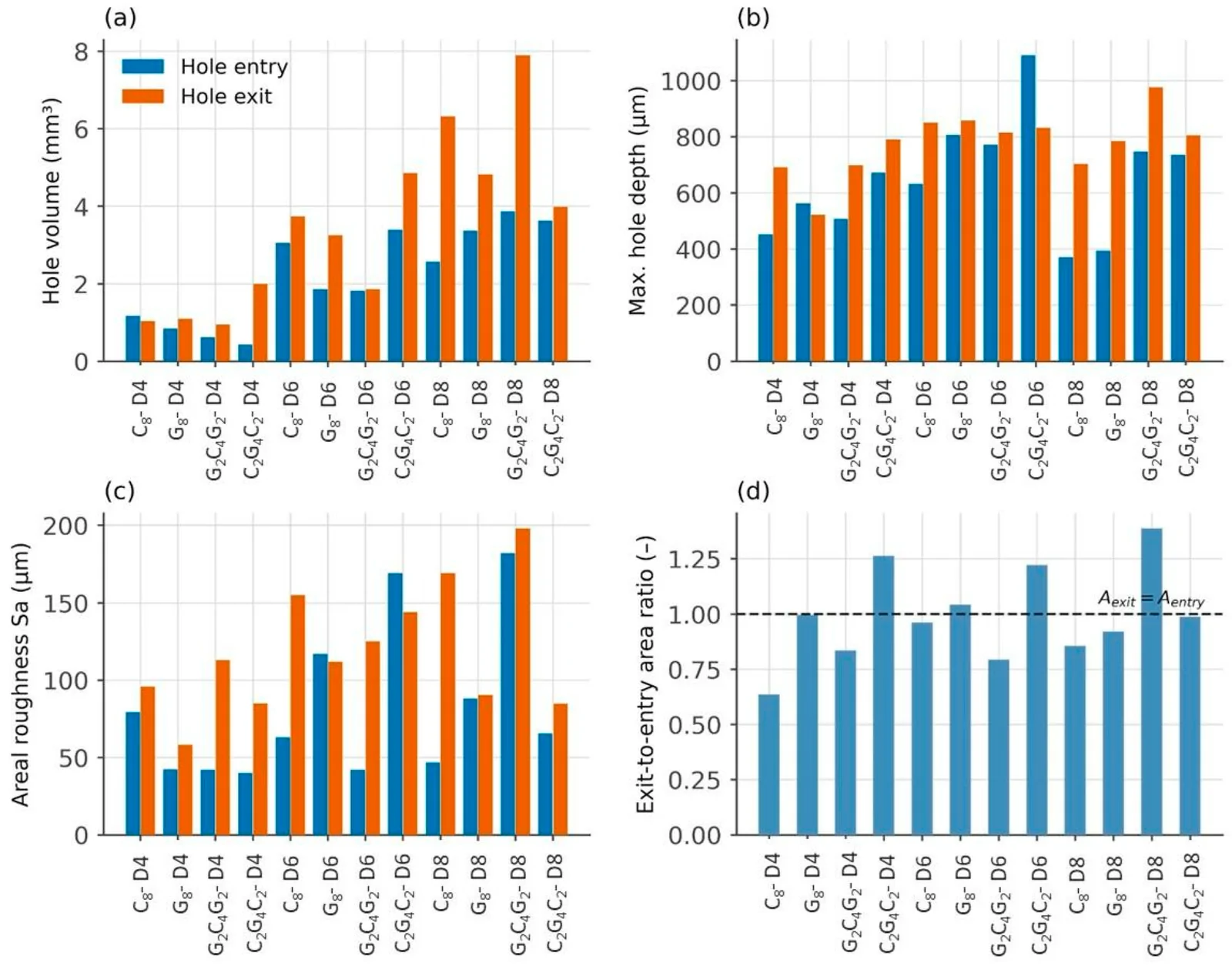
Entry/exit comparison of hole-quality metrics: (**a**) hole-cavity volume, (**b**) maximum hole depth, (**c**) areal surface roughness Sa, and (**d**) exit-to-entry hole surface area ratio (dashed line: symmetric hole, Aexit = Aentry).

**Figure 9:**
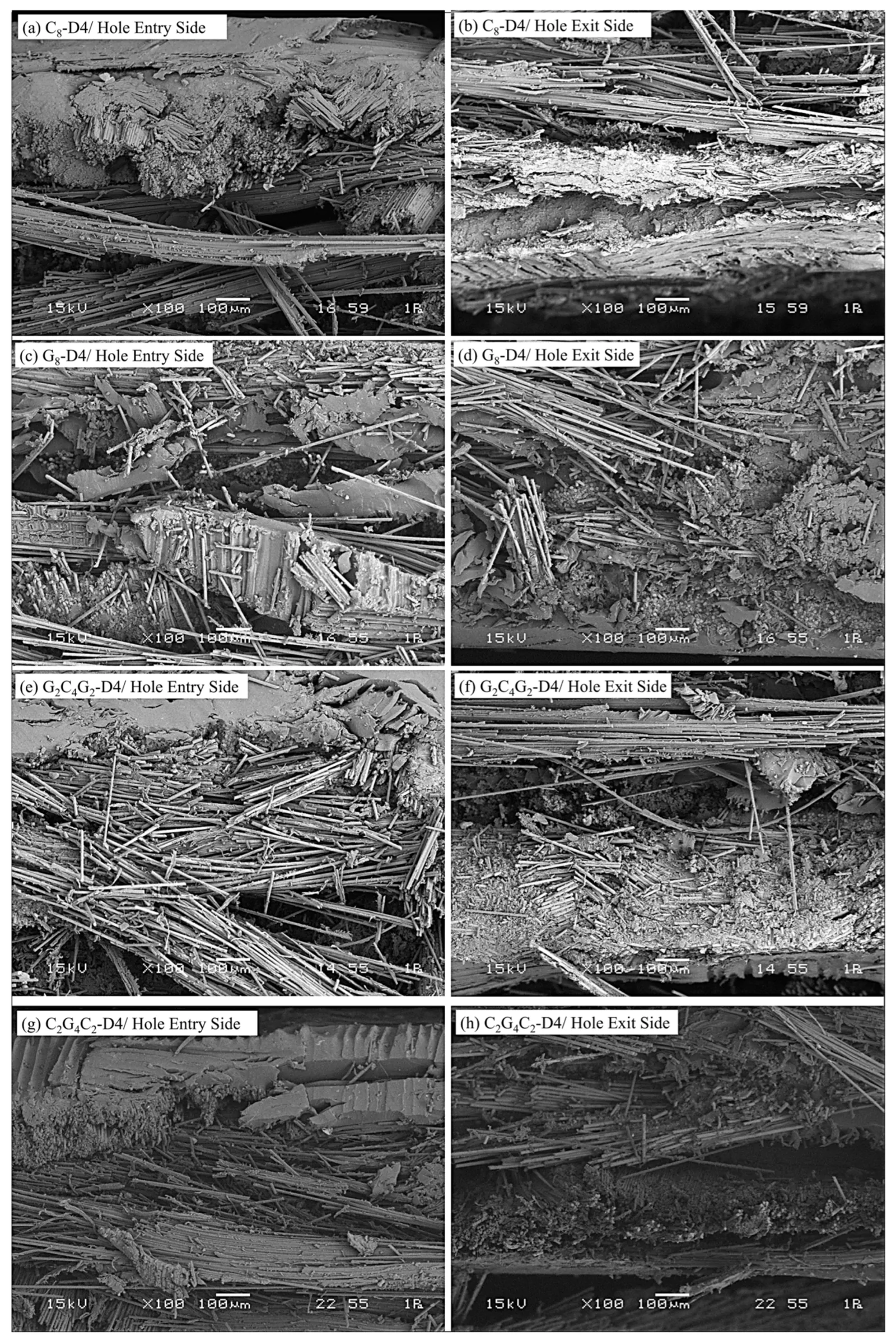
SEM images showing the hole-entry and hole-exit damage morphologies of C_8_, G_8_, G_2_C_4_G_2_, and C_2_G_4_C_2_ laminates drilled using the 4 mm HSS flow step drill.

**Figure 10:**
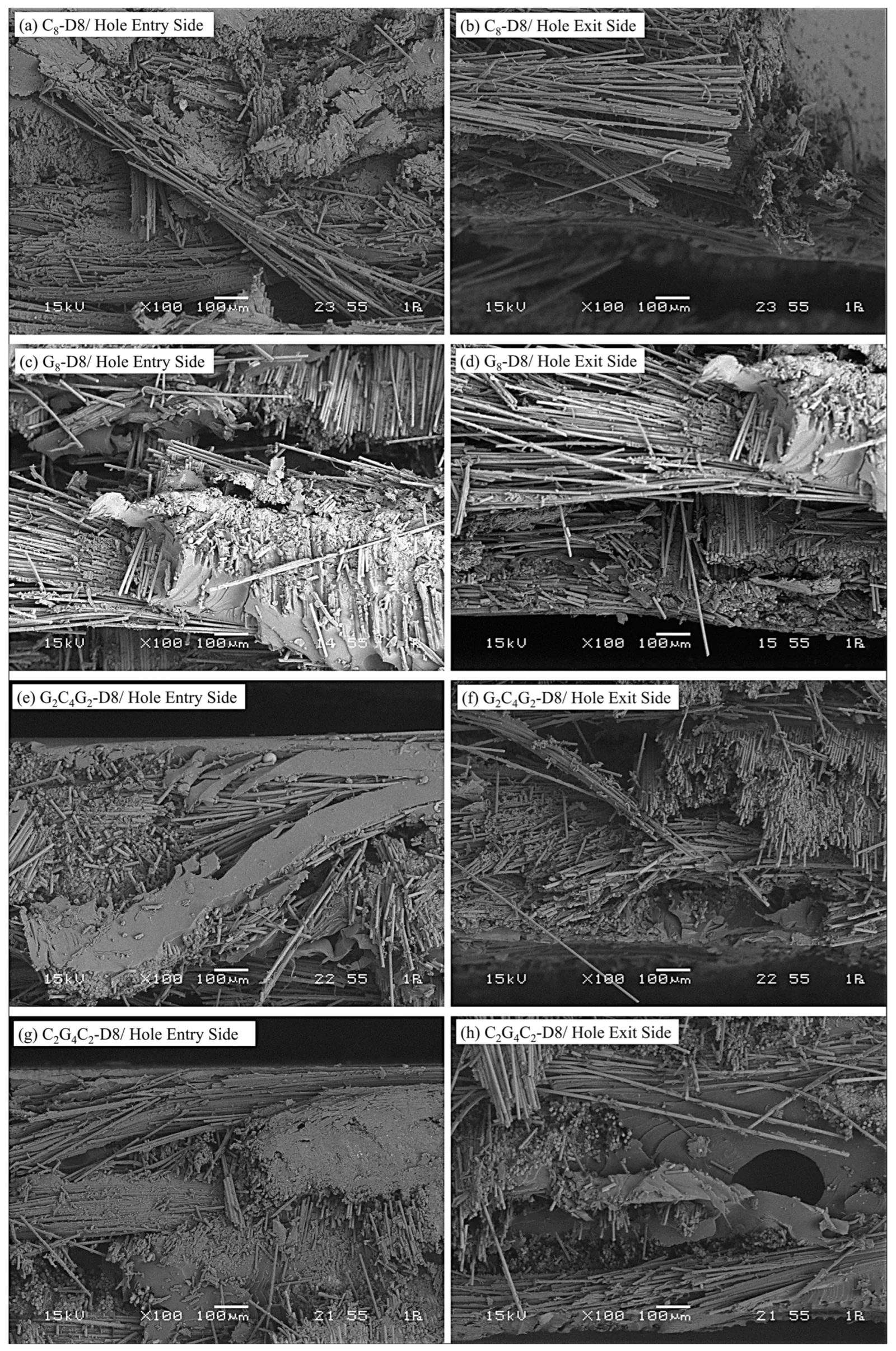
SEM images showing the hole-entry and hole-exit damage morphologies of C_8_, G_8_, G_2_C_4_G_2_, and C_2_G_4_C_2_ laminates drilled using the 8 mm HSS flow step drill.

**Figure 11:**
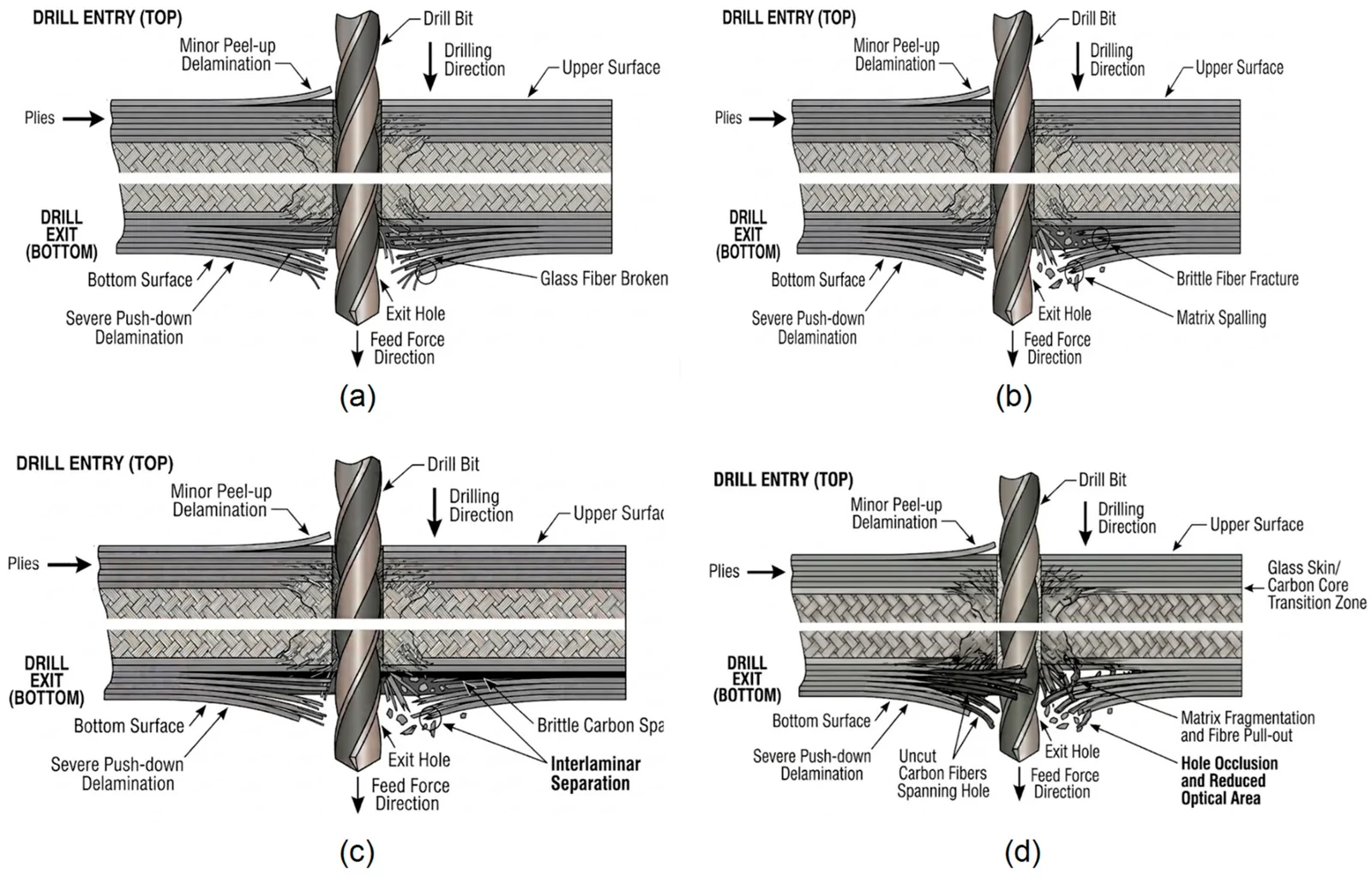
Schematic representation of the characteristic drilling-induced damage mechanisms at hole entry and exit surfaces for (**a**) G_8_, (**b**) C_8_, (**c**) C_2_G_4_C_2_, and (**d**) G_2_C_4_G_2_ composite laminates.

**Figure 12:**
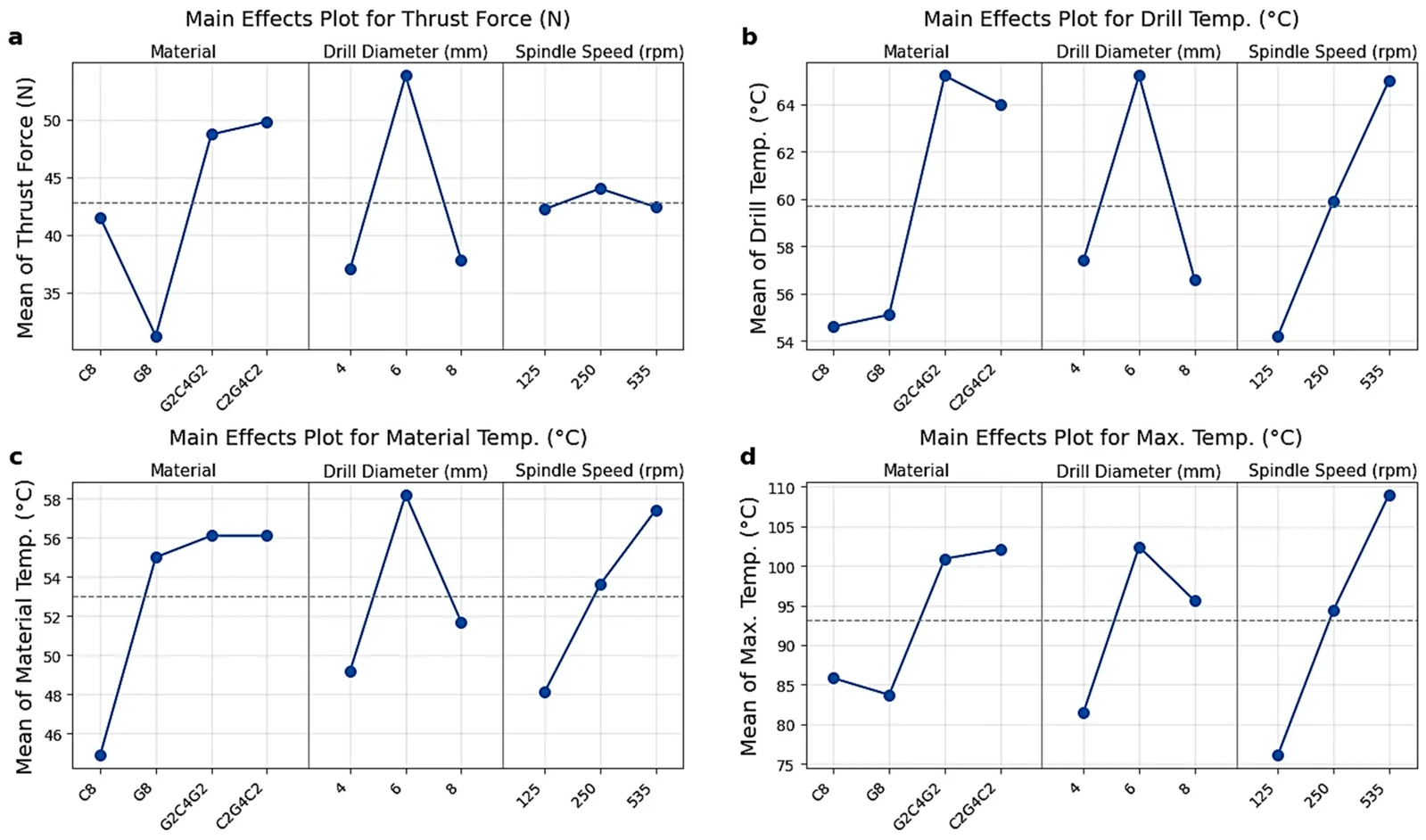
Evaluation of main effects on experimental outputs. The subplots demonstrate the variations in (**a**) thrust force, (**b**) drill temperature, (**c**) material temperature, and (**d**) maximum temperature across different levels of material configurations, drill diameters, and spindle speeds.

**Table 1:** Profilometric hole-quality metrics measured at the hole entry and exit surfaces of the selected specimens (n = 250 rpm).

Specimen	Side	Hole Area (mm^2^)	Sa (µm)	Sz (µm)
C_8_-D4	Entry	8.15	79.4	816
	Exit	5.18	95.9	920
C_8_-D6	Entry	15.6	63.2	1029
	Exit	15	155	1154
C_8_-D8	Entry	35.9	46.8	468
	Exit	30.7	169	1000
G_8_-D4	Entry	6.67	42.5	690
	Exit	6.64	58.1	785
G_8_-D6	Entry	11.8	117	1072
	Exit	12.3	112	1321
G8-D8	Entry	31.5	88.1	526
	Exit	29	90.4	1077
G_2_C_4_G_2_-D4	Entry	6.89	42	672
	Exit	5.75	113	742
G_2_C_4_G_2_-D6	Entry	13.5	42.1	1005
	Exit	10.7	125	1100
G_2_C_4_G_2_-D8	Entry	23	182	982
	Exit	31.9	198	1250
C_2_G_4_C_2_-D4	Entry	4.91	40	892
	Exit	6.2	84.9	1194
C_2_G_4_C_2_-D6	Entry	15	169	1126
	Exit	18.3	144	1276
C_2_G_4_C_2_-D8	Entry	23	65.5	1135
	Exit	22.7	84.7	1195

**Table 2:** Analysis of variance (ANOVA) results detailing sequential sum of squares (Seq SS) and percentage contributions of independent machining parameters and their multi-way interactions on multi-response composite drilling metrics.

Analysis of Variance	Thrust Force (N)			Drill Temp. (°C)		Material Temp. (°C)		Max. Temp. (°C)	
Source	DF	Seq SS	Contribution	Seq SS	Contribution	Seq SS	Contribution	Seq SS	Contribution
Model	35	6430	100.00%	2711	100.00%	2268	100.00%	12,551	100.00%
Linear	7	4165	64.77%	2094	77.24%	1835	80.91%	11,768	93.76%
Material	3	1971	30.65%	855	31.53%	803	35.42%	2533	20.18%
Drill Diameter (mm)	2	2170	33.75%	542	20.00%	511	22.51%	2736	21.80%
Spindle Speed (rpm)	2	24	0.37%	697	25.72%	521	22.98%	6499	51.78%
2-Way Interactions	16	1196	18.59%	465	17.15%	362	15.97%	660	5.26%
Material × Drill Diameter (mm)	6	591	9.19%	204	7.53%	185	8.17%	418	3.33%
Material × Spindle Speed (rpm)	6	300	4.66%	223	8.23%	105	4.62%	194	1.55%
Drill Diameter (mm) × Spindle Speed (rpm)	4	305	4.74%	38	1.39%	72	3.17%	48	0.38%
Error	12	1070	16.63%	152	5.61%	71	3.12%	124	0.99%
Total	35	6430	100.00%	2711	100.00%	2268	100.00%	12,551	100.00%

**Table 3:** Multivariate analysis of variance (MANOVA) results showing Wilks’ Lambda, F-values, and corresponding *p*-values for the effects of material configuration, drill diameter, and spindle speed, along with their two-way interactions, on the combined response vector of thrust force, drill temperature, material temperature, and maximum temperature.

Factor/Interaction	Wilks’ Lambda	F-Value	*p*-Value
Material	0.0925	26.09	<0.0001
Drill Diameter	0.3393	14.52	<0.0001
Spindle Speed	0.2294	22.03	<0.0001
Material × Drill Diameter	0.1451	8.74	<0.0001
Material × Spindle Speed	0.2601	5.57	<0.0001
Drill Diameter × Spindle Speed	0.4969	3.99	<0.0001

## Data Availability

The datasets presented in this article are not readily available because the data are part of an ongoing study. Requests to access the datasets should be directed to the corresponding author.

## Supplementary Materials

The following supporting information can be downloaded at: https://www.mdpi.com/article/10.3390/polym18192316/s1, Table S1: Experimental design layout and measured drilling responses.

## Abbreviations

The following abbreviations are used in this manuscript:

FRP	Fiber-reinforced polymer
CFRP	Carbon fiber-reinforced polymer
GFRP	Glass fiber-reinforced polymer
SEM	Scanning electron microscope
ANOVA	Analysis of variance
Sa	Arithmetical mean height of the surface (ISO 25178-2)
Sz	Maximum height of the surface (ISO 25178-2)
