# Supplementary material for: Thermomechanical Properties of Carbon and Glass Fiber-Reinforced Epoxy Composites as Function of Drilling Parameters
Source: Polymers (Basel). 2026 Sep 22;18(19):2316. doi: 10.3390/polym18192316 (PMC13666827; doi:10.3390/polym18192316)
Supplement: Supplementary file 1 [file polymers-18-02316-s001.zip › polymers-4569387-supplementary.pdf]

Table S1. Experimental design layout and measured drilling responses

Material	Drill Diameter (mm)	Spindle Speed (rpm)	Thrust Force (N)	Drill Temp. (°C)	Material Temp. (°C)	Max. Temp. (°C)
C_8	4	125	27	40.9	33	47.5
C_8	4	250	54	55.7	42.7	67.2
C_8	4	535	40	57.6	45	85.5
C_8	6	125	22	55.2	44.5	79.5
C_8	6	250	54	68.5	56.1	100.6
C_8	6	535	56	68	53.3	116.6
C_8	8	125	53	40.4	34.6	70.9
C_8	8	250	25	50.3	42.6	95.2
C_8	8	535	42	54.9	52	108.6
G_8	4	125	24	53.6	49.8	66.3
G_8	4	250	24	54.3	54.7	76.5
G_8	4	535	25	56.9	57	86.1
G_8	6	125	47	56.4	58.4	79.2
G_8	6	250	42	59.2	62.7	93.5
G_8	6	535	35	62.9	60.9	107.2
G_8	8	125	29	48.5	46.9	69.6
G_8	8	250	27	49	49.5	83.8
G_8	8	535	28	55.2	55	91.6
$G_2C_4G_2$	4	125	40	50	42.9	77
$G_2C_4G_2$	4	250	38	64.7	53.2	89.1
$G_2C_4G_2$	4	535	39	71	59	107.5
$G_2C_4G_2$	6	125	65	66.7	57.2	89.4
$G_2C_4G_2$	6	250	70	67.8	62.9	105
$G_2C_4G_2$	6	535	65	80.9	68.9	125.3
$G_2C_4G_2$	8	125	45	56.2	51.4	81.1
$G_2C_4G_2$	8	250	40	57.7	47.8	110.7
$G_2C_4G_2$	8	535	36	71.2	61.9	122.4
$C_2G_4C_2$	4	125	44	58.7	49.8	73.5
$C_2G_4C_2$	4	250	46	56.1	48.9	94.5
$C_2G_4C_2$	4	535	42	68.9	54.5	106.1
$C_2G_4C_2$	6	125	68	64.3	54.8	88.7
$C_2G_4C_2$	6	250	64	69.7	60.5	112.7
$C_2G_4C_2$	6	535	57	62.6	57.5	130.2
$C_2G_4C_2$	8	125	41	59.9	54	89.9
$C_2G_4C_2$	8	250	43	65.8	60.9	103.2
$C_2G_4C_2$	8	535	43	70	63.6	119.6
